# Regulation of *Botrytis cinerea* Infection and Gene Expression in Cut Roses by Using Nano Silver and Salicylic Acid

**DOI:** 10.3390/plants10061241

**Published:** 2021-06-18

**Authors:** Suong Tuyet Thi Ha, Yong-Tae Kim, Yong Ho Jeon, Hyong Woo Choi, Byung-Chun In

**Affiliations:** 1 Division of Horticulture and Medicinal Plant, Andong National University, Andong 36729, Korea; tuyetsuongha@gmail.com (S.T.T.H.); qkfkadpc@gmail.com (Y.-T.K.); 2 Department of Plant Medicine, Andong National University, Andong 36729, Korea; yongbac@anu.ac.kr (Y.H.J.); hwchoi@anu.ac.kr (H.W.C.)

**Keywords:** cut rose, ethylene signaling, gene expression, gray mold, nano silver, salicylic acid

## Abstract

*Botrytis cinerea* (*B. cinerea*) is one of the necrotrophic pathogens resulting in the heaviest commercial losses in cut rose flowers, and the severity of gray mold disease partly depends on the presence of ethylene during the storage and transport. The effectiveness of nano silver (NS) and salicylic acid (SA) was assessed as a novel control agent in protecting the cut rose flowers against *B. cinerea* infection and ethylene damages. The efficacy of NS and SA was compared with an inoculated control (CON). A non-treated control (NT) was also used to evaluate the natural infection process. The results indicated that pretreatment with 20 mg L^−1^ NS significantly reduced *B. cinerea* growth in rose petals during vase periods. NS effectively suppressed the mRNA levels of ethylene biosynthesis genes (*RhACS2*, *RhACS4*, and *RhACO1*) and the reduction in expression levels of ethylene receptor genes (*RhETR1*, *RhETR2*, and *RhETR5*) and the downstream regulator *RhCTR2* in rose petals after *B. cinerea* inoculation. NS application also decreased the expression of the *B. cinerea snod-prot-like 1* (*Bcspl1*) gene which acts as the virulence factor in cut roses. In NS flowers, the maximum quantum yield of PSII (F_v_/F_m_) value was higher and the leaf temperature was lower on day 1, suggesting that these factors can be used for detecting *B. cinerea* infection and water stress in cut rose flowers. Furthermore, NS improved water relations and extended the vase life of cut rose flowers by 3.3 d, compared with that of NT flowers. In contrast, SA had no inhibitive effects on both *B. cinerea* growth and ethylene response in cut roses. The findings from the present study highlight NS as a promising new candidate for preventing *B. cinerea* infection and ethylene damages and for improving the postharvest quality of cut roses exported overseas.

## 1. Introduction

Roses (*Rosa hybrida* L.) are grown worldwide because of their fragrances, colors, and economic and ornamental values. However, cut rose flowers are prone to infection with gray mold disease which is caused by *B. cinerea* under suitable conditions such as high humidity and low temperature during export and distribution [1]. In rose flowers, *B. cinerea* infection often appears and forms necrotic lesions on the petals and spreads to the entire flower, resulting in failure of the flower opening and petal abscission and complete loss of economic value [1,2,3]. The infection frequently occurs in cut flowers during storage and transport to flower markets, although the petals of cut flowers sometimes do not exhibit disease symptoms at the harvest stage [1]. Additionally, once the rose flowers are infected, the infection rapidly increases and is difficult to control. Therefore, it is important to develop an effective method for suppressing gray mold disease and for improving postharvest quality of cut rose flowers.

The postharvest quality and longevity of cut roses are also influenced by flower senescence which is primarily caused by ethylene synthesis in various floral organs. The perception of ethylene by ethylene receptors is essential for commencing and sustaining the ethylene responses during flower senescence and resistance to diseases [4,5]. A previous study has shown that cut flowers that are susceptible to gray mold disease show considerably increased ethylene synthesis in floral organs, which may contribute to accelerating senescence symptoms in rose flowers [6]. *B. cinerea* infection induces the expression levels of the ethylene biosynthesis genes (*ACS* and *ACO*) in the host plants [7,8]. Another study also indicated that the severity of *B. cinerea* infection symptoms is correlated with ethylene production by leaves and petals in different cut rose cultivars [2,6]. The expression of the *B. cinerea* snod-prot-like 1 (*Bcspl1*) gene, a putative pathogenicity fungal factor, is also enhanced in plants after *B. cinerea* inoculation. In *B. cinerea-* infected plants, the expression of *Bcspl1* is positively correlated with ethylene production [9].

The current literature indicates that chemical fungicides, gamma irradiation, and biological control have been used to control gray mold disease in rose flowers. However, none of these methods have consistently effective control of this necrotrophic pathogen [10,11,12]. Salicylic acid (SA) is one kind of defense-related plant hormone that can induce resistance against many fungal pathogens in plants [13]. Treatment with exogenous SA was found to decrease the incidence of disease and the diameter of lesions caused by *P. expansum, B. cinerea, F. oxysporum,* and *X. axonopodis* in many plants [13]. Nano silver (NS) is a non-residual chemical and is widely used in the medical industry, in water purification, and in vegetable and fruit disinfection. NS is effective against fungal diseases on plants, such as those caused by *B. cinerea* and *Fusarium* sp. [14]. Recently, NS has also been used for extending the postharvest longevity of cut flowers via inhibition of bacteria and ethylene biosynthesis [15,16]. Compared to synthetic fungicides, NS and SA are used for relatively safe evaluation of various plant pathogens [13,14]. However, not much research has been conducted on the effectiveness of NS and SA for treating gray mold disease and the expression of ethylene-related genes in cut roses under export conditions.

In the present study, we examined the effects of NS and SA on *B. cinerea* growth and gene expression in cut rose flowers. We monitored the *B. cinerea* infection rate and the mRNA levels of ethylene biosynthesis, receptor, signaling, and *Bcspl1* genes in rose petals after *B. cinerea* inoculation. Additionally, we also investigated the effectiveness of NS and SA on physiological changes in cut roses after *B. cinerea* infection. Our findings may provide a theoretical basis for developing an effective preservative solution to control the gray mold disease and ethylene damages and for improving postharvest longevity of cut roses during export processes.

## 2. Results

### 2.1. B. cinerea Infection Rate, Vase Life and Flower Opening of Cut Roses

**Figure 1:**
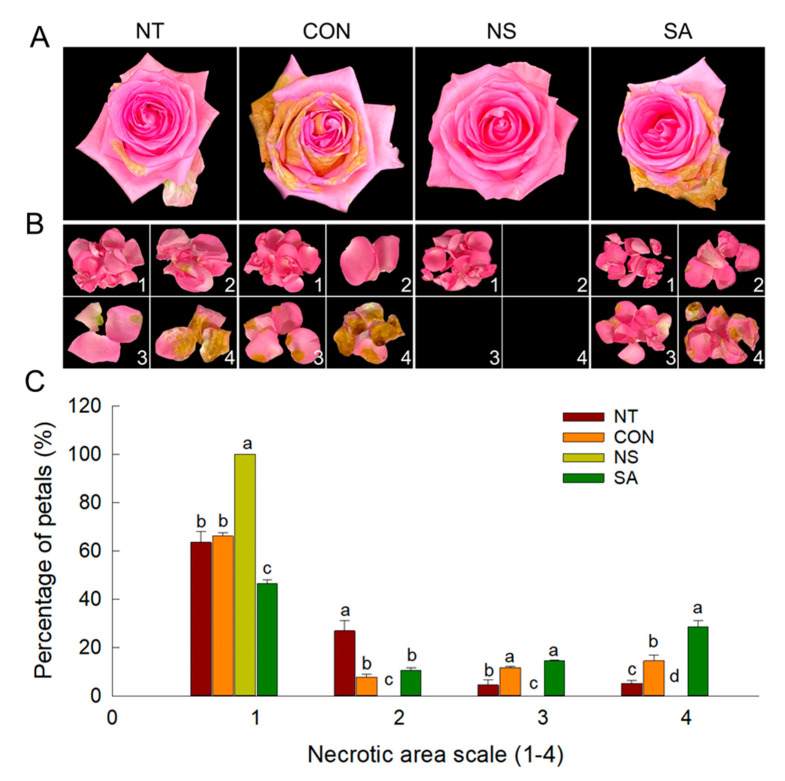
Effect of *B. cinerea* infection on visual appearance (**A**) and the *B. cinerea* infection rate in petals (**B**,**C**) of cut roses on day 7 after the transport simulation. Scale of the extent of the infected rose petal area by *B. cinerea*: 1, no infection; 2, ≤25%; 3, 26–50%; and 4, 51–100%. NT, non-treated flowers; CON, flowers were held in distilled water; NS and SA, cut flowers were treated with nano silver and salicylic acid. CON, NS, and SA flowers were sprayed with *B. cinerea* conidial suspension to induce gray mold development. All flowers were then stored at 10 ± 2°C and 80–90% RH in dark conditions for 4 d for export simulation. Different letters above bars indicate statistically significant differences among treatments at *p* = 0.05 based on Duncan’s multiple range test. Vertical bars show standard errors of the means (*n* = 9).

After *B. cinerea* inoculation and transport simulation, visual symptoms of *B. cinerea* infection on rose plants were recorded on day 1 of vase period in control and SA flowers and on day 5 in NT flowers (data not shown). Control and SA flowers exhibited the highest infection rate (100%) of gray mold disease on rose plants on day 7 of vase life (Table 1). In contrast, 80% of the NT flowers were infected with *B. cinerea* after seven days of the treatment (Table 1). Application of NS significantly decreased the *B. cinerea* infection rate of rose flowers during vase life (Table 1 and Figure 1A).

The lesions in the petals of cut flowers were also recorded and observed daily to assess the effectiveness of NS and SA in inhibiting gray mold disease. The extent of the petal area infected by *B. cinerea* varied according to the treatments. The incidence of infection corresponding to 3 and 4 on the relative scale was higher in control and SA flowers than in NT flowers (Figure 1B,C). The extent of the infected petal area of NS flowers was suppressed during the vase period (Figure 1B,C). These results showed that the NS could completely inhibit gray mold disease on cut rose flowers, whereas SA did not inhibit lesion development.

Early termination of vase life of cut ‘Revival’ roses was due to gray mold disease in control (5.5 d) and SA (6.2 d) treatments (Table 1). Compared to NT flowers (8.6 d), the vase life of NS flowers was significantly prolonged by 3.3 days (Table 1). Interestingly, the maximum flower diameter was largest in NS flowers, whereas it was smaller in control and SA flowers during vase life (Table 1), indicating that the severe gray mold disease on petals caused the failure of full flower opening.

### 2.2. Expression Patterns of Ethylene Biosynthesis and Bcspl1 Genes

**Figure 2:**
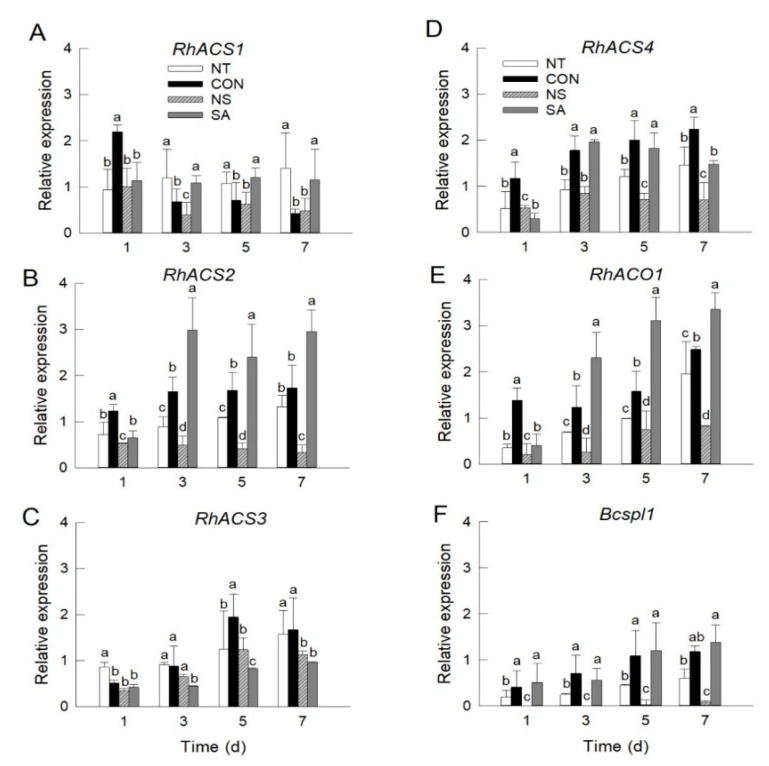
Effect of *B. cinerea* infection on transcript levels of *RhACS1* (**A**), *RhACS2* (**B**), *RhACS3* (**C**), *RhACS4* (**D**), *RhACO1* (**E**), and *Bcspl1* (**F**) in petals of cut roses. mRNA levels of ethylene biosynthesis and *Bcspl1* genes were detected on days 1, 3, 5, and 7 after the transport simulation. NT, non-treated flowers; CON, flowers were held in distilled water; NS and SA, cut flowers were treated with nano silver and salicylic acid. CON, NS, and SA flowers were sprayed with *B. cinerea* conidial suspension to induce gray mold development. All flowers were then stored at 10 ± 2 °C and 80–90% RH in dark conditions for 4 d for export simulation. Different letters above bars indicate statistically significant differences among treatments at *p* = 0.05 based on Duncan’s multiple range test. Vertical bars show standard errors of the means (*n* = 3).

The expression of five ethylene biosynthesis genes and *Bcspl1* in rose petals was detected using quantitative real time PCR (qRT-PCR) on days 1, 3, 5, and 7 after *B. cinerea* inoculation. The mRNA levels of three ethylene biosynthesis genes, *RhACS2*, *RhACS4*, and *RhACO1* were higher in control and SA flowers than in NT and NS flowers (Figure 2B,D,E). The expression of these genes was increased in NT flowers on days 5 and 7; however, there were no changes in NS flowers during vase period (Figure 2B,D,E). The expression of *RhACS3* in petals was induced in all treatments on days 5 and 7 of vase life (Figure 2C). The mRNA levels of *RhACS1* were decreased in control flowers and not affected in other treatments during vase periods. However, the accumulation of *RhACS1* in petals was higher in NT and SA flowers than that of CON and NS flowers on days 3, 5, and 7 (Figure 2A).

A higher level of *Bcspl1* expression was detected in the control and SA flowers during the vase period (Figure 2F). In NT flowers, the expression of *Bcspl1* was enhanced on days 5 and 7 after *B. cinerea* inoculation (Figure 2F). In NS flowers, transcript levels of *Bcspl1* were not detected on days 1 and 3, and remained very low on days 5 and 7 of the vase life (Figure 2F). Among the ethylene biosynthesis genes, *RhACS2*, *RhACS4*, and *RhACO1* exhibited expression patterns similar to those of *Bcspl1* in petals (Figure 2B,D–F), indicating that these genes might be closely correlated with *B. cinerea* growth in roses.

### 2.3. Expression Patterns of Ethylene Receptor and Signaling Genes

**Figure 3:**
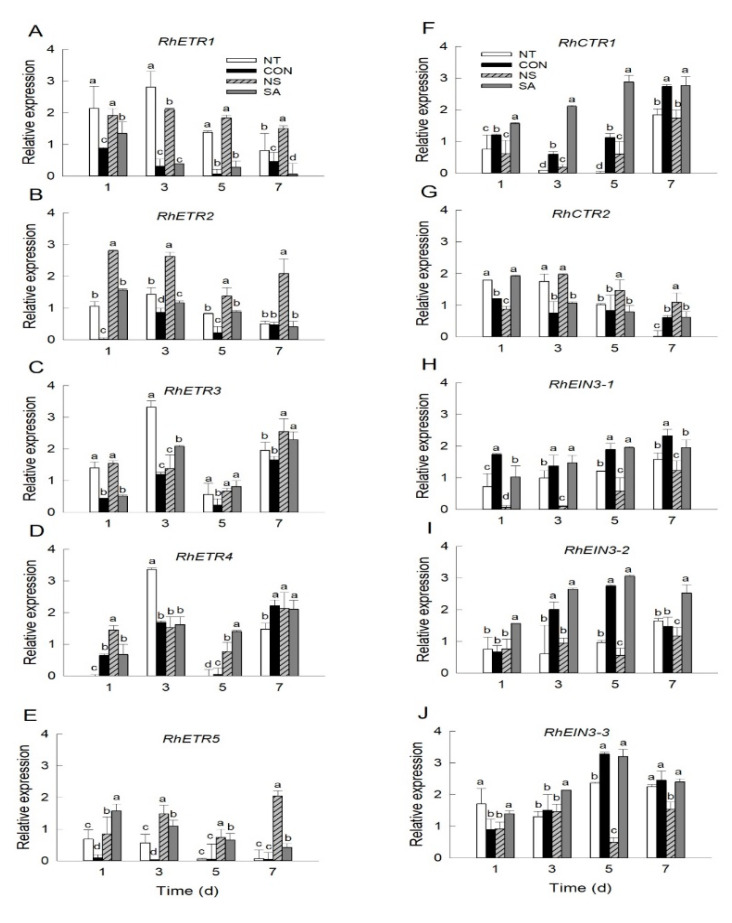
Effect of *B. cinerea* infection on transcript levels of ethylene receptor (**A**–**E**) and signaling (**F**–**J**) genes in petals of cut roses. mRNA levels of ethylene receptor and signaling genes were detected on days 1, 3, 5, and 7 after the transport simulation. NT, non-treated flowers; CON, flowers were held in distilled water; NS and SA, cut flowers were treated with nano silver and salicylic acid. CON, NS, and SA flowers were sprayed with *B. cinerea* conidial suspension to induce gray mold development. All flowers were then stored at 10 ± 2 °C and 80–90% RH in dark conditions for 4 d for export simulation. Different letters above bars indicate statistically significant differences among treatments at *p* = 0.05 based on Duncan’s multiple range test. Vertical bars show standard errors of the means (*n* = 3).

To further assess the effectiveness of NS and SA on gray mold disease and ethylene responses in cut roses, the expression patterns of five ethylene receptors (*RhETR1*-*RhETR5*), two *RhCTRs* (*RhCTR1* and *RhCTR2*), and three *RhEIN3s* (*RhEIN3-1*-*RhEIN3-3*) were determined in the petals. After *B. cinerea* infection, the mRNA levels of three ethylene receptor genes, *RhETR1*, *RhETR2*, and *RhETR5*, and the downstream protein kinase, *RhCTR2* were down-regulated in control and SA flowers during vase life (Figure 3A,B,E,G). In contrast, the decrease in the expression of these genes was suppressed in NS flowers (Figure 3A,B,E,G). In NT flowers, the expression levels of *RhETR1*, *RhETR2*, *RhETR5*, and *RhCTR2* were decreased on days 5 and 7 of the vase period (Figure 3A,B,E,G). The expression of *RhETR3* and *RhETR4* increased during the senescence stage (day 7) in regardless of the treatments (Figure 3C,D). The expression pattern of *RhCTR1* in cut flowers was the opposite of that noted for *RhCTR2* (Figure 3F). In addition, the mRNA levels of *RhCTR1* were higher in control and SA flowers than that of NT and NS flowers (Figure 3F). The *RhEIN3s* expression pattern was similar to that of the ethylene biosynthesis gene *RhACS2*, *RhACS4*, and *RhACO1*. The growth of *B. cinerea* led to a significant increase in the mRNA levels of *RhEIN3s* in the petals of the control and SA flowers (Figure 3H–J). NS treatment effectively suppressed the ethylene-inducible increase in *RhEIN3s* in cut rose flowers (Figure 3H–J).

### 2.4. Changes in the F_v_/F_m_, Leaf Temperature, and Water Relations of Cut Roses

**Figure 4:**
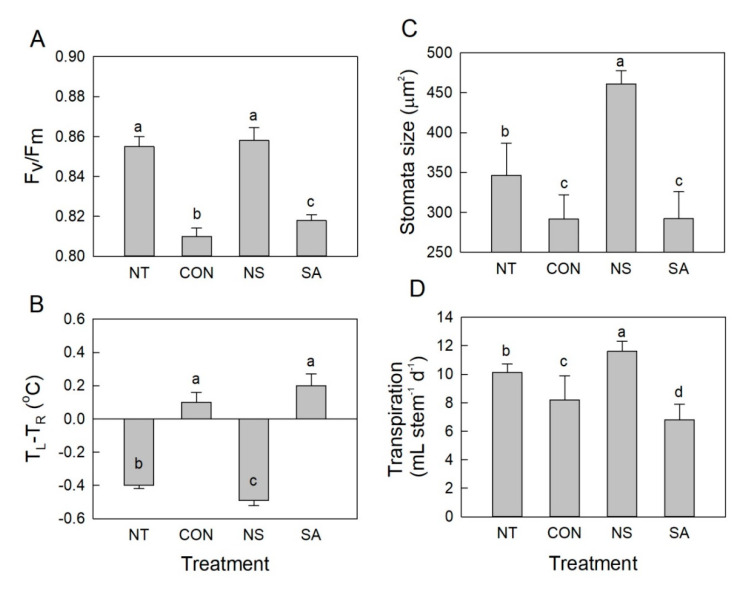
Effect of *B. cinerea* infection on maximum quantum yield of PSII (F_v_/F_m_) (**A**), leaf temperature (**B**), stomata size (**C**), and transpiration (**D**) of cut roses on day 1 after the transport simulation. NT, non-treated flowers; CON, flowers were held in distilled water; NS and SA, cut flowers were treated with nano silver and salicylic acid. Leaf stomatal size and transpiration of cut flowers were measured on day 1 of vase life. CON, NS, and SA flowers were sprayed with *B. cinerea* conidial suspension to induce gray mold development. All flowers were then stored at 10 ± 2 °C and 80–90% RH in dark conditions for 4 d for export simulation. Different letters above bars indicate statistically significant differences among treatments at *p* = 0.05 based on Duncan’s multiple range test. Vertical bars show standard errors of the means (*n* = 3 for (**A**–**C**) and 9 for (**D**)).

The maximum quantum yield of PSII (F_v_/F_m_) was measured using dark-adapted rose leaves, which were subsequently exposed to actinic light on day 1. The *B. cinerea* infection occurring on day 1 decreased the F_v_/F_m_ values in the leaves of control and SA flowers (Figure 4A). The decrease in maximum quantum yield of PSII values was significantly suppressed in NT and NS flowers (Figure 4A). Under light conditions, the temperature difference between the leaf (T_L_) and the reference plate (T_R_) (T_L_ − T_R_) in control and SA flowers on day 1 exhibited positive values (Figure 4B). This finding indicates that the temperature of *B. cinerea*-infected roses increased prior to the appearance of the visual symptoms.

**Figure 5:**
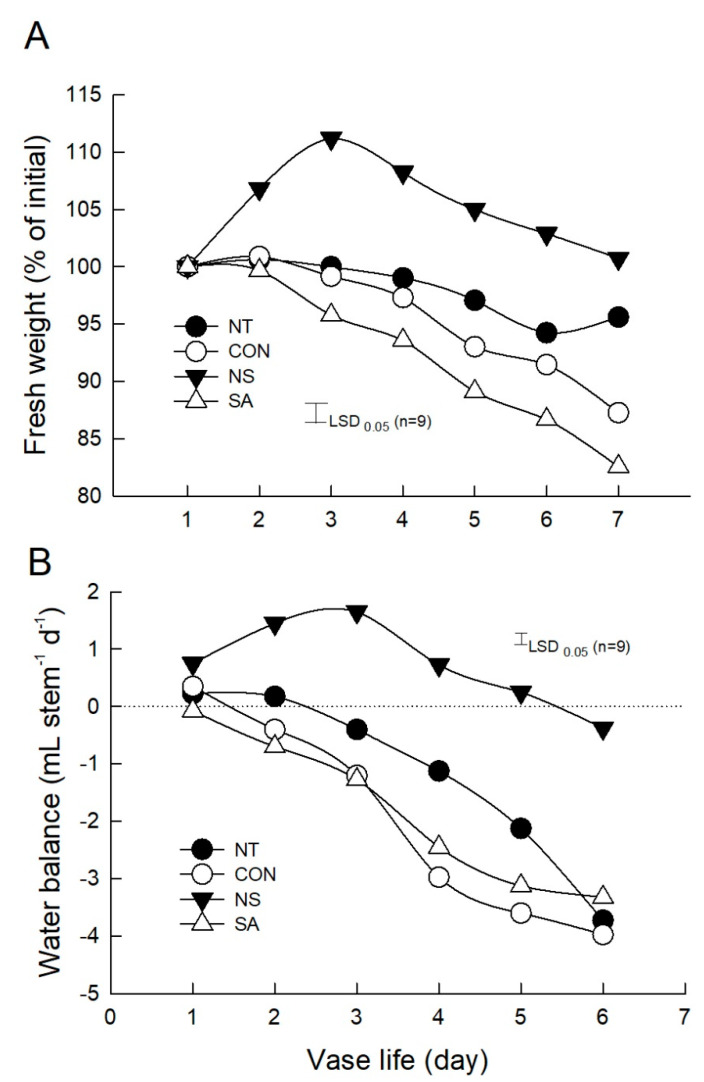
Effect of *B. cinerea* infection on initial fresh weight (**A**) and water balance (**B**) of cut roses. NT, non-treated flowers; CON, flowers were held in distilled water; NS and SA, cut flowers were treated with nano silver and salicylic acid. CON, NS, and SA flowers were sprayed with *B. cinerea* conidial suspension to induce gray mold development. All flowers were then stored at 10 ± 2 °C and 80–90% RH in dark conditions for 4 d for export simulation. Data were presented as the mean ± standard errors (*n* = 9).

Changes in the water relations of the cut flowers were also analyzed daily to evaluate the effect of NS and SA on postharvest quality of cut flowers after *B. cinerea* infection. Under the light conditions, stomatal opening rate and transpiration of control and SA flowers were significantly lower than those of NT and NS flowers (Figure 4C,D). The number of days that flowers retained their initial fresh weight and positive water balance was shortest in control and SA flowers but longest in NS flowers, compared to NT flowers (Figure 5A,B). This result showed that NS not only suppressed the *B. cinerea* growth on cut roses but also effectively improved the water relations of cut flowers during vase period.

## 3. Discussion

Gray mold caused by *B. cinerea*, a necrotrophic pathogen, decreases the economic and ornamental value of cut roses during storage and transportation. After harvest, cut roses are still susceptible to *B. cinerea* infection even under very low temperature conditions because of latent penetration and ability to grow at low temperatures [12]. Thus, early detection and suppression are required to effectively prevent the spread of gray mold in cut flowers.

A previous study showed that the silver ions which are released from nano silver can attack a wide range of biological processes in microorganisms [17]. Therefore, in the current study, NS effectively suppressed the growth of *B. cinerea* spores and prevented the development of the lesions in rose petals during vase life. Previous research showed the effectiveness of SA in inhibiting fungal disease and inducing the disease resistance in several plants [18]. To examine the role of SA in *B. cinerea* resistance, we applied 0.5 mM exogenous SA to cut ‘Revival’ roses and then inoculated the flowers with *B. cinerea*. However, we found no significant difference between the SA-treated flowers and control flowers. Exogenous SA application did not decrease the *B. cinerea* infection rate in rose petals in this study. The postharvest longevity of the cut rose flowers treated with SA was considerably decreased, comparing with NT flowers. SA has resistance effects on various microbial pathogen, but the function of SA in plant defense against the necrotrophic pathogens varies according to plant species [13]. Additionally, the function of SA in the fungal infection process appears to be complex, including at least three strategies: signalization, degradation of SA by the fungal pathogen, and growth inhibition of the pathogen [19].

The development of gray mold disease is related to an increase in ethylene synthesis from the infected tissues [6]. Along with jasmonic acid and SA, ethylene plays an important role in plant defense response to pathogens [20]. When plants are infected with a microorganism, ethylene production by infected tissues is induced for defense against pathogens [8,21]. The results of the present study revealed that the high transcript levels of *RhACS2*, *RhACS4*, and *RhACO1* were associated with the severity of the gray mold disease in rose petals after *B. cinerea* infection. Among ethylene biosynthesis genes in roses, *RhACS1* and *RhACS3* are mainly related to flower development and flower opening, while *RhACS2*, *RhACS4*, and *RhACO1* are responsible for flower senescence and disease defense [22,23]. The increased expression of *RhACS2*, *RhACS4*, and *RhACO1* in control and SA flowers after *B. cinerea* inoculation might lead to endogenous ethylene synthesis and a decrease in mRNA levels of *RhETR1*, *RhETR2*, and *RhETR5* in rose petals. This led to a reduction in the expression level of the downstream protein kinase, *RhCTR2*, thereby resulting in the ethylene response in the cut flowers and acceleration of necrosis development in petals [24]. A previous study also indicated that the expression of both *LeACO1* and *LeACS2* was increased in *B. cinerea*-infected leaves in tomatoes after four days of inoculation and their hybridization intensity was similar to the pattern of *BcactA*, which is a marker for actively growing *B. cinerea* [8]. Simultaneous pretreatment with SA and ethylene has been found to lead to notably increase the transcript levels of *LeACO1* in infected tissues in tomatoes [8]. Consistent with these findings, the present study indicated that the expression of *Bcspl1* was highly induced in control and SA flowers, but strongly inhibited by NS after *B. cinerea* inoculation. In addition, *Bcspl1* exhibited the expression pattern similar to those of *RhACS2, RhACS4*, and *RhACO1* in the petals after *B. cinerea* inoculation. Notably, the expression of *Bcspl1* was strongly induced in the petals in the later stage of the *B. cinerea* infection. It has been well known that the fungal infection was thought to depend on its capacity to kill the host plants and destroy plant tissues. The interaction between *B. cinerea* and host plants is subtle and often evaluated by virulence traits and quantitative susceptibility [25]. The protein encoded by *Bcspl1* might act as an elicitor of plant defense in the early infection phase (penetration and primary lesion formation), whereas it might act as a virulence factor to kill and extract nutrition from host plant tissues during a later phase (plant colonization) [9,26,27]. Moreover, the expression of *Bcspl1* in *B. cinerea* has been found to be induced by ethylene especially during early infection stages and stimulated during later infection stages [9]. In the current study, the increase in mRNA levels of ethylene biosynthesis genes caused by *B. cinerea* infection in rose petals could induce the expression of *Bcspl1*, which acted as the virulence factor and rapidly terminated the vase life of control and SA flowers.

Recently, NS has been applied as an ethylene antagonist and found to interfere with ethylene biosynthesis in many plants [15,16,28]. NS decreased ethylene synthesis by inhibiting the mRNA levels of the ethylene biosynthesis genes, such as *DcACS1, DcACO1, AtACS7,* and *AtACO2* in carnation flowers and *Arabidopsis* seedlings [15,16,28]. The silver ion, which is released from nano silver, can replace the cofactor Cu^2+^ for the ethylene receptor and thereby effectively suppresses ethylene binding [29]. In the current study, the suppression of ethylene action by NS could prevent the degradation of ethylene receptors and the downstream protein kinase by maintaining the mRNA levels of these genes, resulting in the inhibition of the ethylene responses and the necrosis development in rose petals.

Disease infection often causes disorder of physiological metabolism in the infected plant tissues. The stomatal opening rate, leaf temperature, maximum quantum yield of PSII of leaves, and water relations of the control and SA flowers were negatively affected by *B. cinerea* infection. Stomata can be considered to participate in the plant innate immune responses via regulation of stomatal closure. Bacteria and fungi can trigger stomatal closure through pathogen-associated molecular patterns, which prevents penetration through these pores [30,31,32]. Thus, the *B. cinerea* infection could induce stomatal closure in control and SA flowers to prevent infiltration through stomatal pores. Stomatal closure reduced the transpiration rate in the leaves of the cut flowers and led to an increased leaf temperature. Our results are consistent with a previous study showing that the infection of *Pseudoperonospora cubensis* caused an increase in leaf temperature via the regulation of stomatal transpiration in cucumbers [33]. Interaction of SA and abscisic acid was found to be required for stomata responses to disease infection in plants [34]. SA is also a potent inducer of stomatal closure [32]. These previous findings strongly supported our results indicating that flowers treated with SA exhibited a smallest stomatal opening rate, lowest transpiration, and highest leaf temperature after *B. cinerea* inoculation.

Among the different chlorophyll fluorescence parameters, F_v_/F_m_ is the most important value that is used as a plant stress indicator, since it declines under most stress conditions and reflects the maximum photochemical efficiency [35,36,37,38]. Decrease in F_v_/F_m_ is often observed before the visual appearance of disease or stress symptoms; therefore F_v_/F_m_ has been used to predict and detect those abnormal stress status in many plant species [36,37,39]. A highly significant negative correlation was observed between leaf temperature and F_v_/F_m_ in peanut and cotton under stress conditions, suggesting that higher photodamage in the photosynthetic apparatus of peanut due to increase in leaf temperature [37]. The reduction in F_v_/F_m_ in control and SA flowers indicated that downregulation in maximum quantum yield of PSII was induced in the tissues by *B. cinerea* infection at an early stage. This finding coincided with an increase in leaf temperature, which was likely due to stomatal closure. Consistent with these findings, previous studies also observed the considerable decrease in F_v_/F_m_ in tobacco plants infected with *B. cinerea* [40], maize and banana plants infected with *Fusarium* [41], and in melon infected by *Dickeya dadantii* [41]. Our results also revealed that the F_v_/F_m_ value and leaf temperature can be used for detecting infection of plant tissues by *B. cinerea* at an early stage of the infection process.

In summary, our results indicated that the *B. cinerea* growth on rose petals considerably increased the expression of ethylene biosynthesis genes, leading to accumulation of the virulence factor *Bcspl1. B. cinerea* infection also decreased the transcript levels of ethylene receptor genes and of the downstream regulator *RhCTR2*, thereby inducing the plant responses to ethylene. The growth of the *B. cinerea* caused the downregulation in maximum quantum yield of PSII via regulation of stomatal function and leaf temperature. Nano silver significantly inhibited *B. cinerea* growth and suppressed the ethylene responses in rose petals by inhibiting the expression of ethylene biosynthesis and signaling genes. Moreover, nano silver effectively maintained the F_v_/F_m_ in leaves, improved water relations, and reduced the incidence of senescence of cut flowers, thereby prolonging the vase life of cut roses. Thus, our results show that nano silver can be a promising chemical for preventing *B. cinerea* infection and ethylene damages, and for improving the postharvest quality of cut roses that are exported overseas.

## 4. Materials and Methods

### 4.1. Plant Materials

Cut roses (*Rosa hybrida* L. cv ‘Revival’) were harvested at commercial maturity stage from a commercial greenhouse in Daegu, Korea in June 2020. Rose plants were provided with a nutrient solution including calcium, nitrogen, phosphorus, magnesium, potassium, and other trace substances. Changes in environmental conditions in the greenhouse such as light density, relative humidity (RH), and air temperature were monitored during the year using data loggers. After harvest, the cut flowers were kept in buckets containing tap water and transferred to the laboratory within 2 h for further treatments.

### 4.2. Preparation of B. cinerea Conidial Suspension

*B. cinerea* used in this study was obtained from Agricultural Genetic Resource Information Center, Korea. Prior to treatments, the isolated *B. cinerea* conidia were transferred and grown on potato dextrose agar media at 25 °C for 14 days. To collect the *B. cinerea* conidia, 25 mL sterile distilled water was added to the culture petri dishes and gently filtered by using four layers of sterile gauze. The concentration of *B. cinerea* conidia was determined using a hemocytometer, and the final concentration was adjusted to 10⁵ conidia mL^−1^ for the experiments.

### 4.3. Nano Silver, Salicylic Acid, and B. cinerea Treatments

Based on our previous study [42], cut rose flowers were placed and sprayed with 20 mg L^−1^ NS (ShangHai HuZheng Nano Tech Co., Ltd., ShangHai, China) or 0.5 mM SA (Daejung Chemicals, Korea). NT and CON flowers were placed in distilled water without treatment solutions. CON, NS, and SA flowers were inoculated with the *B. cinerea* conidial suspension (10⁵ conidia mL^−1^) to induce gray mold development, whereas NT flowers were not inoculated with *B. cinerea* and used to assess the natural infection process. Subsequently, all cut flowers were held at 10 ± 2 °C and 80–90% RH under dark conditions for 4 days for simulation of export conditions. After the transport simulations, cut flowers were trimmed to 50 cm of the length along with three upper leaves. Each cut rose was placed in a glass vase containing 350 mL distilled water. For vase life evaluation, the flowers were maintained in a controlled environmental room at 25 ± 1 °C, 50–55% relative humidity, and a photoperiod of 12 h with light supplied by fluorescent tubes at 20 µmol m^−2^ s^−1^ light intensity.

### 4.4. Evaluation of B. cinerea Infection and the Morphological and Physiological Characteristics of Cut Flowers

Number of rose stems with flower and leaf damage and visual symptoms of *B. cinerea* in rose petals were recorded daily to evaluate the effectiveness of NS and SA on inhibition of *B. cinerea* infection in cut roses. The extent of development of visual symptoms of *B. cinerea* was assessed on a relative scale of 1–4 according to the extent of the infected rose petal area, as follows: 1, no infection; 2, ≤25%; 3, 26–50%; and 4, 51–100%. The vase life of cut roses was considered terminated when cut flowers showed *B. cinerea* growth on petals that corresponded to 3 or 4 points on the relative scale. Other senescence symptoms, such as bent neck, bluing, or wilting of leaf and flower were also observed when the cut flowers reached the senescent stage.

Changes in morphological and physiological characteristics of cut flowers such as vase life, flower opening, fresh weight, and water uptake were also determined daily at 9:30 am. The water balance of cut flowers was determined by deducting the daily transpiration rate from daily water uptake. Leaf stomatal sizes were measured under light conditions (after 45 min at 20 mol^−2^ s^−1^ light intensity) on day 1 of the vase life. Stomata were obtained from the abaxial surface of the uppermost leaf of the cut flowers using Suzuki’s Universal Micro-Printing method. The stomatal width and length were measured by using the Image J software (Version 1.49p, NIH, Bethesda, MD, USA) and the stomatal size was determined using the equation:

S = π × r_1_ × r_2_ (1)

where S is stomatal size, r_1_ is half of the width, and r_2_ is half of the length.

### 4.5. Measurement of Leaf Temperature and Chlorophyll Fluorescence (CF) Parameters

The uppermost leaves of the cut flowers were used for measuring the leaf temperature and CF parameters. The CF parameters of these leaves were measured using the FluorPen FP110/D device (Photon Systems Instruments, Brno, Czech Republic). A dark-adapted leaf was obtained by covering the leaf with a leaf-clip for 15 min. The clip was then opened and the minimal fluorescence (F_0_) and maximal fluorescence (F_m_) of the leaf were measured. The maximal quantum efficiency of PSII (F_v_/F_m_) of the leaf was automatically calculated from the F_0_ and F_m_ values.

To measure the temperature of the rose leaves, thermal images of cut rose flowers were obtained using a thermal camera (FLIR, VUE™ PRO R, FLIR systems) after morphological measurement. For each treatment, thermal images of cut flowers were taken under the similar conditions. To reduce the effect of distance variation on the results, the distance between the rose leaves and the camera lens was set as 30 cm for all flowers during the measurements. A reference plate (10 cm × 10 cm × 3 mm) was also placed at the same height as the leaf measured. Thermal images of the cut roses were exported to the computer by using FLIR tools for measuring the leaf temperature. The temperature difference between the leaf (T_L_) and the reference plate (T_R_) was calculated as T_L_ − T_R_.

### 4.6. RNA Isolation and cDNA Synthesis

Petals were detached from cut rose flowers after the transport simulation on days 1, 3, 5 and 7. These rose petals were immediately frozen in liquid nitrogen and then stored at −80 °C until RNA extraction. The rose petals were ground with liquid nitrogen to a fine powder (200 mg) using a prechilled mortar and pestle and total RNA was isolated from the fine powder by using the GeneJET plant RNA Purification Mini Kit (Thermo Fisher Scientific Baltics UBA V.A. Graiciuno, Vilnius, Lithuania) with a slight modification of the manufacturer’s protocol. The total RNA was quantified at 260 nm/280 nm using a Nanodrop spectrophotometer (NanoDrop 2000, Thermo Scientific, Waltham, MA, USA). Total RNA (0.1 µg) was used to synthesize the first strand of cDNA using Power cDNA Synthesis Kit (INTRON Biotechnology, Inc., Gyeonggi-do, Republic of Korea) as per the manufacturer’s instruction, in a Bio-Rad PTC-100 Programmable Thermal Controller (MJ Research Inc., Hercules, CA, USA).

### 4.7. qRT-PCR

The mRNA levels of ethylene biosynthesis, receptor, and signaling and *B. cinerea* snod-prot-like 1 (*Bcspl1*) genes in rose petals were detected using the BIO-RAD CFX Connect Real-Time System (Life Science, Hercules, CA, USA). The sequences of forward and reverse primers used for qRT-PCR are listed in Table 2. The gene-specific primers were designed by an online version of Primer 3 software, and then synthesized by Cosmogenetech (Seoul, Korea). *Rosa hybrida* actin (*RhACT1*) and *B. cinerea* actinA (*BcactA*) genes were used as an internal control to confirm the amount of template RNA. The reaction mixtures (20 µL) contained 5 µL cDNA as a template, 10 µL iQ™ SYBR^®^ Green Supermix (Life Science, Hercules, CA, USA), 1 µL forward and reverse primers (10 µM), and 4 µL water. The qRT-PCR reactions were conducted using the following PCR program: 95 °C for 60 s for pre-denaturation (one cycle) and followed by 40 cycles of 95 °C for 10 s (denaturation), 65 °C for 10 s (annealing), and 72 °C for 40 s (extension). The final threshold cycle value was the mean of three independent biological replications and the coefficient of variance for each gene was also calculated. The relative level of the gene expression was measured as the absolute integrated absorbency normalized to the relative actin.

### 4.8. Statistical Analysis

The vase life and disease incidence data were designed with nine replicates for each treatment and one flower per replication. qRT-PCR analysis, stomatal size, CF parameters, and leaf temperature measurements were performed with three biological replicates. All data were presented as mean ± standard error (SE). One-way analysis of variance was conducted for data. When significant effects were detected, post hoc pairwise comparisons of group means were executed with Duncan’s multiple range test, with a significance level of *p* = 0.05. Least significant difference (LSD; *p* = 0.05) values were calculated for mean separation using the critical values of *t* for two-tailed tests. Data analysis was performed by using SPSS version 22.0 (IBM, Somers, NY, USA).

## Figures and Tables

**Table 1:** Infection rate, vase life, and flower opening of cut rose flowers after *B. cinerea* inoculation.

Treatment	*B. cinerea* Incidence (%) ˣ	Vase Life (Days)	Maximum Flower Diameter (%) ʸ
Non-treatment (NT)	80.0 ± 0.0 b ᶻ	8.6 ± 0.3 b	121.1 ± 1.3 ab
Control (CON)	100.0 ± 0.0 a	5.5 ± 0.4 c	109.3 ± 1.9 c
Nano silver (NS)	0.0 ± 0.0 c	11.9 ± 0.8 a	125.7 ± 2.3 a
Salicylic acid (SA)	100 ± 0.0 a	6.2 ± 0.4 c	108.6 ± 5.8 c

ˣ The incidence of *B. cinerea* in cut roses was evaluated on day 7 of vase life. ʸ Maximum flower diameter (percentage of the initial diameter) was measured and compared among the treatments on day 4 of vase life. ᶻ Different letters among the treatments indicate the statistically significant differences at *p* = 0.05 based on Duncan’s multiple range test (*n* = 9).

**Table 2:** Primer sequences used in qRT-PCR for gene analysis in rose petals.

Gene (Accession Number)	Forward Primer	Reverse Primer	Size
*RhACS1* (AY378152.1)	5′-CAGTGAGAAAGGGGAGCTTG-3′	5′-TGTATTGAACCGGGATGGTT-3′	102
*RhACS2* (AY803737.1)	5′-GCGAACAGGGGTACAACTTC-3′	5′-GGGTTTGAGGGGTTGGTAAT-3′	147
*RhACS3* (AY803738.1)	5′-CAGTGAGAAAGGGGAGCTTG-3′	5′-AACCATCCCGGTTCAATACA-3′	142
*RhACS4* (AY525068.1)	5′-GCTTCCAACTTGGGATCAAA-3′	5′-GCTCCATGAAACTTGCCATT-3′	100
*RhACO1* (AF441282.1)	5′CGTTCTACAACCCAGGCAAT-3′	5′-TTGAGGCCTGCATAGAGCTT-3′	130
*RhETR1* (AY953869.1)	5′-TGACTGGCCTGATGTCTCTG-3′	5′-GGCAACTGGTGAAAAGGAAA-3′	158
*RhETR2* (AF127220.1)	5′-CTGCGTTAGAGCAGCAACTG-3′	5′-GGAATTCGGCGATATCTTCA-3′	131
*RhETR3* (AY953392.1)	5′-CCATGAGTTGAAAGGGAGGA-3′	5′-GGCTCACCAAAATCACCACT-3′	156
*RhETR4* (AF159172.1)	5′-TTGAAGTCGTTGCAGACCAG-3′	5′-TCATGACAGCAAGGAAGTCG-3′	168
*RhETR5* (AF441283.1)	5′-TGTGTGGAGCGACACATCTT-3′	5′-TGAGGGCAGTAGCACATGAC-3′	120
*RhEIN3-1* (AF443783)	5′-TGCTGAAGATGATGGAGGTG-3′	5′-GCAGGGCCATTCTTATCAAA-3′	142
*RhEIN3-2* (AY919867.1)	5′-ATTGAACTTGGCCAATCAGG-3′	5′-GCAGTCATCTTGTCCTGCAA-3′	168
*RhEIN3-3* (KC484653.1)	5′-GCCAGTGGATCTTTGGTGAT-3′	5′-ACTTGAAGCCCTTCCCTCAT-3′	149
*RhCTR1* (AY032953.1)	5′-GGCTCTGATGTTGCTGTGAA-3′	5′-CAAGTTTGGGGGCTTTGTAA-3′	150
*RhCTR2* (AY029067.1)	5′-CGAGCAACCCCACTATTGTT-3′	5′-TTATGTTTCAAGCGCGACAG-3′	109
*RhACT1* (KC514918.1)	5′-GTTCCCAGGAATCGCTGATA-3′	5′-ATCCTCCGATCCAAACACTG-3′	116
*Bcspl1* (XM024691684.1)	5′-CCTACGACGTTGGCTACGAT-3′	5′-CCTCAAGAACTTCCCCAACA-3′	123
*BcactA*(XM024697950.1)	5′-GCACCACCCGAGAGAAAATA-3′	5′-AAGAGTACGACGAGTCCGGA-3′	169

## Data Availability

All data generated and analyzed during this study are included in this published article.

## References

[1] Williamson B., Duncan G.H., Harrison J.G., Harding L.A., Elad Y., Zimand G. (1995). Effect of humidity on infection of rose petals by dry-inoculated conidia of *Botrytis cinerea*. Mycol. Res..

[2] Elad Y., Volpin H. (1988). The involvement of ethylene and calcium in gray mold of pelargonium, ruscus, and rose plants. Phytoparasitica.

[3] Muñoz M., Faust J.E., Schnabel G. (2019). Characterization of *Botrytis cinerea* From Commercial Cut Flower Roses. Plant Dis..

[4] Borochov A., Woodson W. (2011). Physiology and Biochemistry of Flower Petal Senescence. Hortic. Rev..

[5] Pantelides I.S., Tjamos S.E., Paplomatas E.J. (2010). Insights into the role of ethylene perception in tomato resistance to vascular infection by *Verticillium dahliae*. Plant Pathol..

[6] Elad Y. (1988). Involvement of ethylene in the disease caused by Botrytis cinerea on rose and carnation flowers and the possibility of control. Ann. Appl. Biol..

[7] Benito E.P., ten Have A., van’t Klooster J.W., van Kan J.A.L. (1998). Fungal and plant gene expression during synchronized infection of tomato leaves by Botrytis cinerea. Eur. J. Plant Pathol..

[8] Díaz J., ten Have A., van Kan J.A.L. (2002). The Role of Ethylene and Wound Signaling in Resistance of Tomato to *Botrytis cinerea*. Plant Physiol..

[9] Chagué V., Danit L.V., Siewers V., Schulze-Gronover C., Tudzynski P., Tudzynski B., Sharon A. (2006). Ethylene sensing and gene activation in *Botrytis cinerea*: A missing link in ethylene regulation of fungus-plant interactions?. Mol. Plant Microbe Interact. Mpmi.

[10] Elad Y., Shtienberg D. (1995). Botrytis cinerea in greenhouse vegetables: Chemical, cultural, physiological and biological controls and their integration. Integr. Pest Manag. Rev..

[11] Chu E.-H., Shin E.-J., Park H.-J., Jeong R.-D. (2015). Effect of gamma irradiation and its convergent treatment for control of postharvest *Botrytis cinerea* of cut roses. Radiat. Phys. Chem..

[12] Williamson B., Tudzynski B., Tudzynski P., van Kan J.A. (2007). *Botrytis cinerea*: The cause of grey mould disease. Mol. Plant Pathol..

[13] Koo Y.M., Heo A.Y., Choi H.W. (2020). Salicylic Acid as a Safe Plant Protector and Growth Regulator. Plant Pathol. J..

[14] Kim S.W., Jung J.H., Lamsal K., Kim Y.S., Min J.S., Lee Y.S. (2012). Antifungal Effects of Silver Nanoparticles (AgNPs) against Various Plant Pathogenic Fungi. Mycobiology.

[15] Naing A.H., Win N.M., Han J.S., Lim K.B., Kim C.K. (2017). Role of Nano-silver and the Bacterial Strain Enterobacter cloacae in Increasing Vase Life of Cut Carnation ‘Omea’. Front. Plant Sci..

[16] Park D.Y., Naing A.H., Ai T.N., Han J.-S., Kang I.-K., Kim C.K. (2017). Synergistic Effect of Nano-Sliver with Sucrose on Extending Vase Life of the Carnation cv. Edun. Front. Plant Sci..

[17] Dakal T.C., Kumar A., Majumdar R.S., Yadav V. (2016). Mechanistic Basis of Antimicrobial Actions of Silver Nanoparticles. Front. Microbiol..

[18] Hua L., Yong C., Zhanquan Z., Boqiang L., Guozheng Q., Shiping T. (2018). Pathogenic mechanisms and control strategies of Botrytis cinerea causing post-harvest decay in fruits and vegetables. Food Qual. Saf..

[19] Dieryckx C., Gaudin V., Dupuy J.W., Bonneu M., Girard V., Job D. (2015). Beyond plant defense: Insights on the potential of salicylic and methylsalicylic acid to contain growth of the phytopathogen *Botrytis cinerea*. Front. Plant Sci..

[20] Wu L., Huang Z., Li X., Ma L., Gu Q., Wu H., Liu J., Borriss R., Wu Z., Gao X. (2018). Stomatal Closure and SA-, JA/ET-Signaling Pathways Are Essential for *Bacillus amyloliquefaciens* FZB42 to Restrict Leaf Disease Caused by Phytophthora nicotianae in *Nicotiana benthamiana*. Front. Microbiol..

[21] Bleecker A.B., Kende H. (2000). Ethylene: A gaseous signal molecule in plants. Annu. Rev. Cell Dev. Biol..

[22] Ma N., Cai L., Lu W., Tan H., Gao J. (2005). Exogenous ethylene influences flower opening of cut roses (*Rosa hybrida*) by regulating the genes encoding ethylene biosynthesis enzymes. Sci. China. Ser. C Life Sci..

[23] In B.-C., Ha S.T.T., Lee Y.S., Lim J.H. (2017). Relationships between the longevity, water relations, ethylene sensitivity, and gene expression of cut roses. Postharvest Biol. Technol..

[24] Lund S.T., Stall R.E., Klee H.J. (1998). Ethylene regulates the susceptible response to pathogen infection in tomato. Plant Cell.

[25] Veloso J., Van Kan J.A.L. (2018). Many shades of grey in *Botrytis*-Host plant interactions. Trends Plant Sci..

[26] Tudzynski P., Sharon A. (2003). Fungal pathogenicity genes. Appl. Mycol. Biotechnol..

[27] Wolpert T.J., Dunkle L.D., Ciuffetti L.M. (2002). Host-selective toxins and avirulence determinants: What’s in a name?. Annu. Rev. Phytopathol..

[28] Syu Y.-Y., Hung J.-H., Chen J.-C., Chuang H.-W. (2014). Impacts of size and shape of silver nanoparticles on Arabidopsis plant growth and gene expression. Plant Physiol. Biochem..

[29] Rodríguez F.I., Esch J.J., Hall A.E., Binder B.M., Schaller G.E., Bleecker A.B. (1999). A copper cofactor for the ethylene receptor ETR1 from Arabidopsis. Science.

[30] Melotto M., Zhang L., Oblessuc P.R., He S.Y. (2017). Stomatal Defense a Decade Later. Plant Physiol..

[31] Melotto M., Underwood W., He S.Y. (2008). Role of stomata in plant innate immunity and foliar bacterial diseases. Annu. Rev. Phytopathol..

[32] Gudesblat G.E., Torres P.S., Vojnov A.A. (2009). Stomata and pathogens: Warfare at the gates. Plant Signal. Behav..

[33] Oerke E.-C., Steiner U., Dehne H.-W., Lindenthal M. (2006). Thermal imaging of cucumber leaves affected by downy mildew and environmental conditions. J. Exp. Bot..

[34] Prodhan M.Y., Munemasa S., Nahar M.N.-E.-N., Nakamura Y., Murata Y. (2018). Guard Cell Salicylic Acid Signaling Is Integrated into Abscisic Acid Signaling via the Ca^2+^/CPK-Dependent Pathway. Plant Physiol..

[35] Rolfe S.A., Scholes J.D. (2010). Chlorophyll fluorescence imaging of plant-pathogen interactions. Protoplasma.

[36] Shahenshah, Isoda A. (2010). Effects of Water Stress on Leaf Temperature and Chlorophyll Fluorescence Parameters in Cotton and Peanut. Plant Prod. Sci..

[37] Rousseau C., Belin E., Bove E., Rousseau D., Fabre F., Berruyer R., Guillaumès J., Manceau C., Jacques M.-A., Boureau T. (2013). High throughput quantitative phenotyping of plant resistance using chlorophyll fluorescence image analysis. Plant Methods.

[38] Kim J.H., Bhandari S.R., Chae S.Y., Cho M.C., Lee J.G. (2019). Application of maximum quantum yield, a parameter of chlorophyll fluorescence, for early determination of bacterial wilt in tomato seedlings. Hortic. Environ. Biotechnol..

[39] Pineda M., Soukupová J., Matouš K., Nedbal L., Barón M. (2008). Conventional and combinatorial chlorophyll fluorescence imaging of tobamovirus-infected plants. Photosynthetica.

[40] Rossi F.R., Krapp A.R., Bisaro F., Maiale S.J., Pieckenstain F.L., Carrillo N. (2017). Reactive oxygen species generated in chloroplasts contribute to tobacco leaf infection by the necrotrophic fungus *Botrytis cinerea*. Plant J. Cell Mol. Biol..

[41] Kuckenberg J., Tartachnyk I., Noga G. (2009). Temporal and spatial changes of chlorophyll fluorescence as a basis for early and precise detection of leaf rust and powdery mildew infections in wheat leaves. Precis. Agric..

[42] Ha S.T.T., Kim Y.T., In B.C. (2020). Assessment of the preservative solutions for reducing *Botrytis cinerea* infection in cut roses. Flower Res. J..

